# The Impact of Japan's 2004 Postgraduate Training Program on Intra-Prefectural Distribution of Pediatricians in Japan

**DOI:** 10.1371/journal.pone.0077045

**Published:** 2013-10-30

**Authors:** Rie Sakai, Wei Wang, Norihiro Yamaguchi, Hiroshi Tamura, Rei Goto, Ichiro Kawachi

**Affiliations:** 1 Department of Global Health and Population, Harvard School of Public Health, Boston, Massachusetts, United States of America; 2 Department of Medical Education, Juntendo University School of Medicine, Tokyo, Japan; 3 Department of Pediatrics and Adolescent Medicine, Juntendo University School of Medicine, Tokyo, Japan; 4 Department of Medicine, Brigham and Women's Hospital, Boston, Massachusetts, United States of America; 5 Department of Medicine, Harvard Medical School, Boston, Massachusetts, United States of America; 6 Department of Medicine, Division of Hematology/Oncology, Beth Israel Deaconess Medical Center, Boston, Massachusetts, United States of America; 7 Department of Ophthalmology and Visual Sciences, Kyoto University Graduate School of Medicine, Kyoto, Japan; 8 Solutions Center for Health Insurance Claims, Kyoto University Hospital, Kyoto, Japan; 9 Department of Epidemiology, Harvard School of Public Health, Boston, Massachusetts, United States of America; 10 Hakubi Center of Advanced Research, Kyoto University, Kyoto, Japan; 11 Graduate School of Economics, Kyoto University, Yoshida, Sakyo, Kyoto, Japan; 12 Department of Society, Human Development, and Health, Harvard School of Public Health, Boston, Massachusetts, United States of America; Tokyo Metropolitan Institute of Medical Science, Japan

## Abstract

**Objective:**

Inequity in physician distribution poses a challenge to many health systems. In Japan, a new postgraduate training program for all new medical graduates was introduced in 2004, and researchers have argued that this program has increased inequalities in physician distribution. We examined the trends in the geographic distribution of pediatricians as well as all physicians from 1996 to 2010 to identify the impact of the launch of the new training program.

**Methods:**

The Gini coefficient was calculated using municipalities as the study unit within each prefecture to assess whether there were significant changes in the intra-prefectural distribution of all physicians and pediatricians before and after the launch of the new training program. The effect of the new program was quantified by estimating the difference in the slope in the time trend of the Gini coefficients before and after 2004 using a linear change-point regression design. We categorized 47 prefectures in Japan into two groups: 1) predominantly urban and 2) others by the definition from OECD to conduct stratified analyses by urban-rural status.

**Results:**

The trends in physician distribution worsened after 2004 for all physicians (p value<.0001) and pediatricians (p value = 0.0057). For all physicians, the trends worsened after 2004 both in predominantly urban prefectures (p value = 0.0012) and others (p value<0.0001), whereas, for pediatricians, the distribution worsened in others (p value = 0.0343), but not in predominantly urban prefectures (p value = 0.0584).

**Conclusion:**

The intra-prefectural distribution of physicians worsened after the launch of the new training program, which may reflect the impact of the new postgraduate program. In pediatrics, changes in the Gini trend differed significantly before and after the launch of the new training program in others, but not in predominantly urban prefectures. Further observation is needed to explore how this difference in trends affects the health status of the child population.

## Background

Optimizing the distribution of physicians poses a challenge to the health systems of many countries [1]–[15]. The maldistribution of physicians may arise along different geographical dimensions, such as between urban and sparsely populated rural areas or between areas of medical specialization.

In Japan, attempts have been made to increase medical student quotas and the number of medical schools in order to increase the numbers of physicians. Thus far, however, these efforts have not resolved the disparity in the distribution of physicians [4], [13].

The Ministry of Health, Labor and Welfare (MHLW) introduced a new postgraduate medical education program to improve residency training in 2004. The new scheme was introduced to address deficiencies in clinical training in the country's 6-year undergraduate medical program. The generally accepted view in the Japanese media is that access to medical care has become more unequal, particularly since the advent of the new postgraduate training program [16], [17]. The new program includes two years of mandatory post-graduate training focused on primary care in designated clinical training hospitals. Before this program, most new medical graduates underwent postgraduate training at a hospital associated with the university from which they graduated. Under the new program, new medical graduates select urban hospitals for their training rather than rural university hospitals [18]. Consequently, the number of physicians in university hospitals has decreased. Most of the hospitals in Japan have ties to university hospital departments, and physicians are dispatched to hospitals under the supervision of a particular university department. Because the number of physicians at university hospitals has decreased, university hospitals find it increasingly difficult to send physicians to affiliated hospitals, which are often located in rural areas. Furthermore, physicians working in rural areas return to university hospitals to fill the void left by the lack of new medical graduates. The Japan Medical Association Research Institute reported that almost 80% of all university hospitals have reduced the dispatch of physicians to other medical institutions since the launch of the new training program [16]. However, studies on the impact of the new post-graduate training system on physician distribution in Japan have been limited [4], [19]–[21].

The situation in pediatrics in Japan is particularly serious. There have been reports of pediatricians' deaths due to overwork and suicides resulting from depression due to overwork [22]. Moreover, there are regions without pediatricians, forcing parents and guardians to travel long distances to obtain treatment for sick children. Medical practitioners and patients alike are adversely affected by the shortage of pediatricians.

There is a need to address the distribution of physicians according to geographic and specialization needs [16]–[18]. However, previous investigations have been limited to a focus on physician totals [1], [2], [4], [13], [23] with one notable study by Ehara focusing on the number of pediatricians in Japan [21].

In this study, trends in the distribution of pediatricians as well as total numbers of physicians in Japan from 1996 to 2010 were examined to identify the impact of the launch of the new training program in 2004 on physician, especially pediatrician, distribution in Japan.

## Methods

The Gini coefficient was used to assess the distribution of physicians, as in several previous studies [2], [4], [13], [23], [24]. The Gini coefficient is traditionally used to analyze the distribution of income and wealth and has a theoretical range from 0 (perfect evenness) to 1 (maximum possible unevenness). It provides a standardized value to reflect the relative unevenness of distribution. The Gini coefficient is calculated by a method described elsewhere [2], [4], [13], [23], [23,24].

Japan has three levels of government: municipal, prefectural and national. Municipalities are the basic geographical units of administration. Prefectures and municipalities in Japan are roughly comparable to states and counties in the United States. There are 47 prefectures in Japan. Data on the number of all physicians and pediatricians by municipality level were obtained from the Survey of Physicians, Dentists, and Pharmacologists [25], which is conducted by the MHLW every two years. All licensed physicians must complete this survey and register their working address and specialty according to the Medical Practitioners Law [26]. The estimated registry rate is reported to be between 87% and 90% [27]. Data from 1996 to 2010 were publicly available online at the time of this study. Therefore, the following eight time points were used for the analysis: 1996, 1998, 2000, 2002, 2004, 2006, 2008 and 2010. In this study, pediatricians are defined as physicians whose main specialty is pediatrics, and we did not include physicians who provide pediatric care although their main specialty is not pediatrics.

Data on municipal populations were obtained from the Basic Resident Registers, which are collected and compiled by the Ministry of Internal Affairs and Communications in March of each year [28].

To calculate the Gini coefficient for all physicians, the general population of each locality was used (i.e. plotting the Lorenz curve which is based on the cumulative proportion of the total population served by physicians within each locality). The x-axis of the Lorenz curve represents the cumulative proportion of total population ranked by physician-to-population ratio and the y-axis represents that of total physicians. To calculate the Gini coefficient for pediatricians, the child population in each locality was used. The x-axis of the Lorenz curve represents the cumulative proportion of child population ranked by pediatrician-to-child population ratio and the y-axis represents that of pediatricians. The child population is defined as the population under 15 years old because children in this age group are treated by pediatricians in Japan.

This study is composed of the following four steps. First, the trends in the Gini coefficient are shown for all of Japan using prefectures as the study unit. Second, the trends in the Gini coefficient are analyzed for all of Japan using municipalities as the study unit. Third, the Gini coefficient is calculated using municipalities as the study unit within each prefecture to assess whether there are significant changes in inequity in the distribution of all physicians and pediatricians before and after the launch of the new training program. The effect of the 2004 postgraduate training program is quantified by estimating the difference in the slope in the time trend of the physician Gini using a linear change-point regression procedure. We fit the following model for the study outcome:
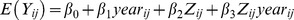
where E(Y) is the expected value of the dependent variable, which is the Gini coefficient, and z is defined as a function that equals 1 when year _ij_ ≥2004 and is otherwise equal to 0. When expressed in terms of the mean response prior to and after 2004,



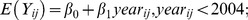


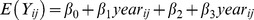


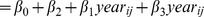


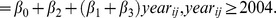



Thus, β_3_ provides a measure of the difference in the trend in E (Y) prior to and after the year 2004 and can be interpreted as the effect of the new postgraduate medical education program. Hereafter, the *pre-period* is defined as the period from 1996 to 2002 and the *post-period* is defined as the period from 2004 to 2010 because the survey in 2004 reflected the impact of the new postgraduate medical education program since the new program was introduced on April 1, 2004 and the Survey of Physicians, Dentists, and Pharmacologists is conducted in December every year. Fourth, stratified analyses by urban/rural status were conducted to explore the impact of the launch of a new postgraduate medical education program in 2004 in urban and rural areas. To classify 47 prefectures into urban or rural status, we employed the definition from Organization for Economic Co-operation and Development (OECD regional typology). The definition from OECD classifies regions into predominantly Urban, Intermediate and Predominantly Rural by prefecture level in Japan. In our study, we categorized prefectures in Japan into two groups: 1) predominantly urban and 2) others [29]. Because no standard definition of the term “rural” exists [30]–[33], we also conducted a series of robustness checks. Previous studies have employed one of the following definitions [30]: 1) metropolitan statistical area [32], [34], which is comparable to metropolitan area codes in Japan: 2) population size [13], [30], [32]: and 3) population density [30], [35]. In this study, we used the following two definitions for robustness checks. First, we employed the definition of the metropolitan area code by the Ministry of Internal Affairs and Communications. Metropolitan areas outside of Tokyo consist of central cities (cities with a population of 500,000 or more) as well as surrounding municipalities where 1.5% or more of the population commutes to the central cities. Tokyo has 23 wards, each of which is considered a central city, although some of the wards have population of less than 500,000 [36]. The Ministry of Internal Affairs and Communications classifies municipalities into the following five categories: 1) central cities for major metropolitan areas, 2) surrounding municipalities of central cities for major metropolitan areas, 3) central cities for metropolitan areas, 4) surrounding municipalities of central cities for metropolitan areas, and 5) other municipalities. In this study, the prefectures that include central cities for major metropolitan areas are defined as urban, and others are defined as rural. Second, we employed population density to define urban/rural status as an alternate definition. Under this alternative definition, the prefectures with population density more than 1000/km^2^ are defined as urban, and others are defined as rural. Japan underwent administrative re-organization by a large-scale merging of municipalities. Therefore, the total number of municipalities dramatically decreased during the study period. The number of physicians and the population of the municipality in every data set were adjusted for the new municipal boundaries, by merging former smaller municipality into later larger ones. To examine the trend in the geographic distribution of physicians using the Gini coefficient, the number and boundaries of geographic units must be fixed. Therefore, the 2010 boundaries (n = 1,750) were used for all time points.

A two-tailed p value of less than 0.05 was considered statistically significant. All analyses were performed using SAS software 9.2 (SAS Institute, Inc., Cary, NC).

## Results

Table 1 shows the total population, child population, number of total physicians, pediatricians, per capita total physicians and per child capita pediatricians between 1996 and 2010. The child population decreased, the number of pediatricians increased, and the number of per child capita pediatricians increased over the study period (Table 1).

**Table 1:** Trend in total population, child population, number of total physician and pediatricians, and per 100,000 capita total physician and per 100,000 child capita pediatricians.

	1996	1998	2000	2002	2004	2006	2008	2010
Total population (*1,000,000)	124.9	125.6	126.1	126.5	126.8	127.1	127.1	127.1
Child population (*1,000,000)	19.7	19.1	18.6	18.1	17.8	17.5	17.3	17.1
Number of total physicians	230,297	236,933	243,201	249,574	256,668	263,540	271,897	280,431
Number of pediatricians	13,781	13,989	14,160	14,481	14,677	14,700	15,236	15,870
Per capita total physicians	184.4	188.7	192.9	197.3	202.4	207.4	214.0	220.7
Per child capita pediatricians	70.3	73.2	76.3	79.9	82.5	83.8	88.1	93.0

Table 2 shows trends in the Gini coefficient for Japan using prefectures and municipalities (Table 2). The Gini coefficients using both prefectures and municipalities as the units of analysis show little change during the study period.

**Table 2:** Gini coefficient using prefecture, municipality as study units.

	1996	1998	2000	2002	2004	2006	2008	2010
Physician totals								
Gini coefficient by prefecture	0.12	0.12	0.12	0.12	0.12	0.11	0.11	0.11
Gini coefficient by municipality	0.34	0.34	0.33	0.33	0.33	0.33	0.33	0.33
Pediatrician								
Gini coefficient by prefecture	0.13	0.12	0.12	0.12	0.12	0.12	0.11	0.11
Gini coefficient by municipality	0.38	0.37	0.37	0.37	0.36	0.36	0.36	0.37

**Figure 1:**
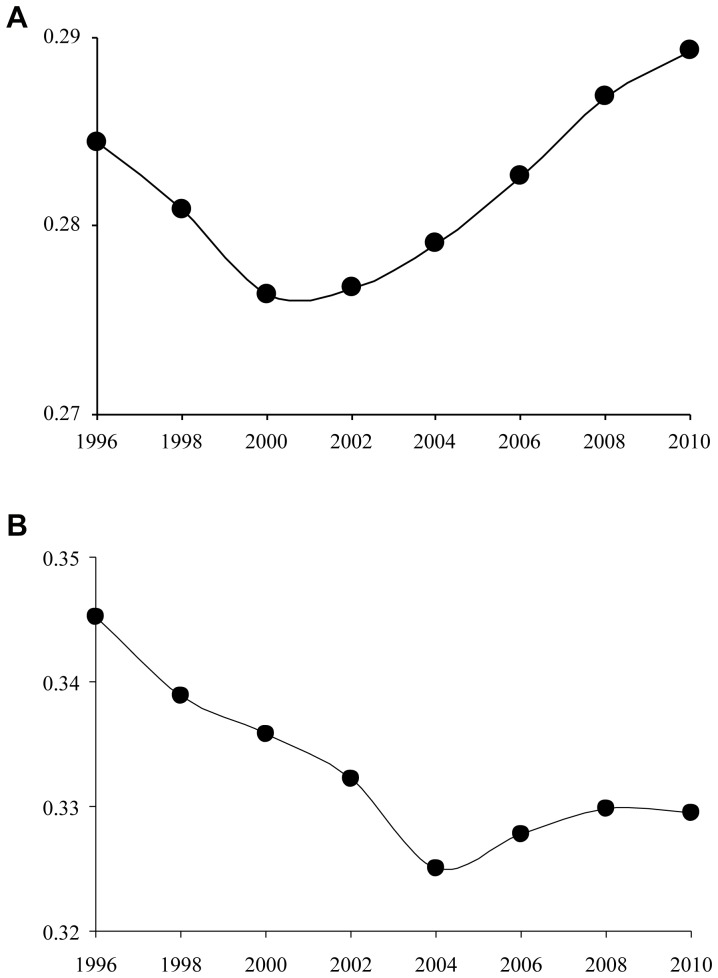
Figure 1-a; Mean of Gini coefficient of all physicians in intra-prefectural distributions. Figure 1-b; Mean of Gini coefficient of pediatricians in intra-prefectural distributions.

Figure 1 shows the mean of the Gini coefficient using municipalities as the unit of analysis each year for all physicians (Figure 1-a) and pediatricians (Figure 1-b). The linear change-point regression model shows (Table 3) that there was a statistically significant change (improvements in distribution) in the Gini coefficient in the *pre-period* for all physicians (p-value  = 0.0002) and pediatricians (p-value  = 0.0042), and the changes statistically differed between the *pre-* and the *post-period* for all physicians (p-value <.0001) as well as pediatricians (p-value  = 0.0057), i.e. deterioration in *the post-period*. This result suggests that intra-prefectural distribution of physicians worsened because the Gini coefficients using both prefectures and municipalities as the units of analysis showed little change during the study period.

**Table 3:** Results of linear change-point regression models for intra-prefectural distributions.

Effect		Estimate	SE^a^	p value
all physicians				
β0	intercept	0.2838	0.00916	<.0001
β1	year	−0.0028	0.00073	0.0002
β2	z^b^	−0.0185	0.0043	<.0001
β3	z ^b^•year	0.00625	0.00103	<.0001
pediatrician				
β0	intercept	0.3444	0.01035	<.0001
β1	year	−0.0042	0.00146	0.0042
β2	z^b^	−0.0248	0.00865	0.045
β3	z ^b^•year	0.00575	0.00207	0.0057

a: SE: standard error.

b: Z: a function that equals 1 when year _ij_ >2004 and 0 otherwise.

**Figure 2:**
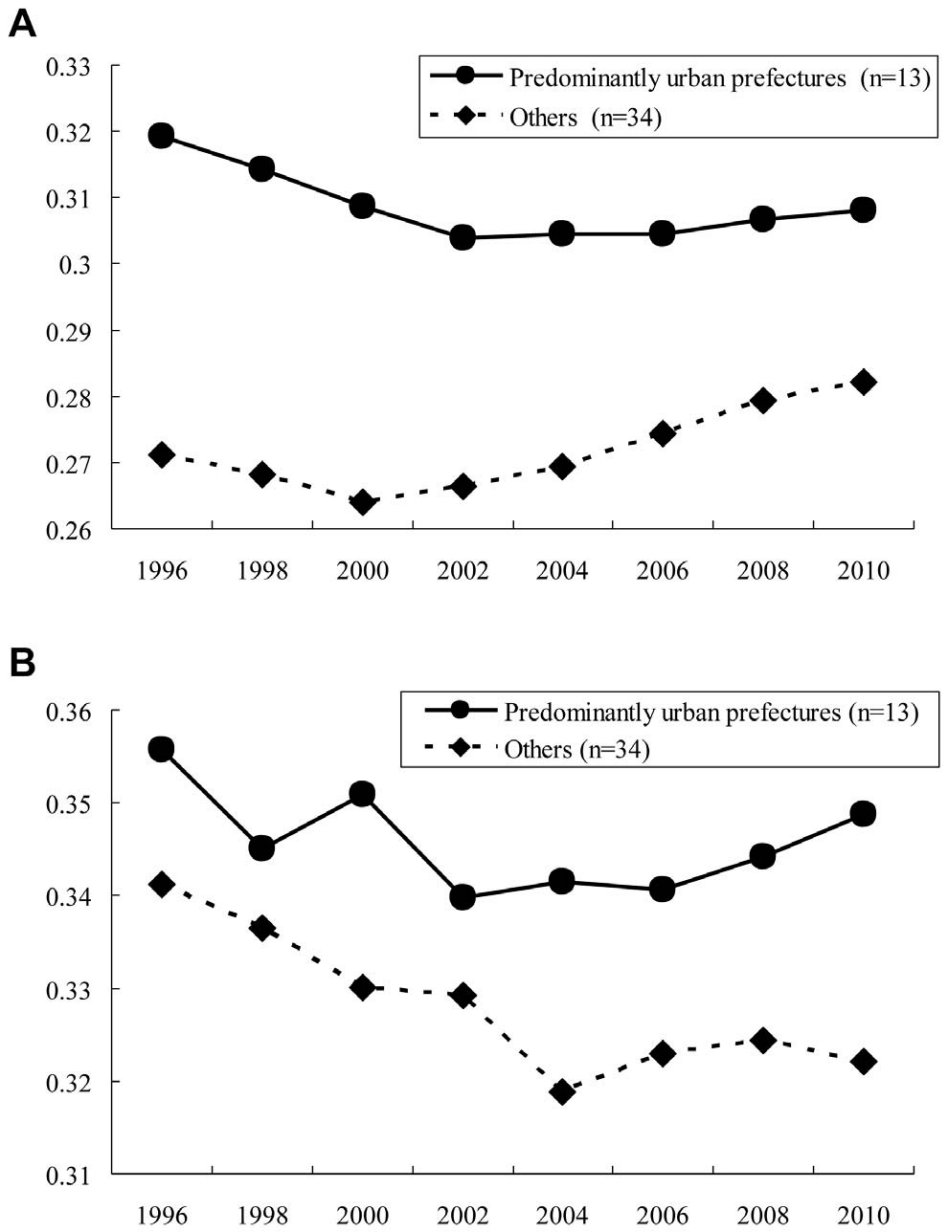
Figure 2-a Mean Gini coefficients of all physicians in intra-prefectural distributions; Prefectures were classified into predominantly urban prefectures and others according to the definition of OECD. Figure 2-b Mean Gini coefficients of pediatricians in intra-prefectural distributions; Prefectures were classified into predominantly urban prefectures and others according to the definition of OECD.

A total of 13 prefectures (Miyagi, Saitama, Chiba, Tokyo, Kanagawa, Shizuoka, Aichi, Kyoto, Osaka, Hyogo, Nara, Hiroshima, and Fukuoka) were defined as predominantly urban prefectures and the remaining 34 are defined as others. In 2010, 58.4% of the total population of Japan lived in predominantly urban prefectures, and the remaining of 41.6% lived in others. Figure 2 shows the mean of the Gini coefficient stratified by predominantly urban prefectures and others for all physicians (Figure 2-a) and pediatricians (Figure 2-b). The mean of the Gini coefficient in predominantly urban prefectures is higher for all physicians and pediatricians during the study period. The stratified analyses by predominantly urban prefectures and others (Table 4) shows that for all physicians, predominantly urban and others showed the same trends; there is a statistically significant decrease in the Gini in the *pre-period* (*β_1_* = −0.00510, p-value  = 0.0003 in predominantly urban prefectures and *β_1_* = −0.0019, p-value  = 0.0201 in others) and there is a significant change in trends for the worse between the *pre-* and the *post-period* (*β_3_* = 0.00645, p-value  = 0.0012 in predominantly urban prefectures and *β_3_* = 0.00617, p-value <0.0001) For pediatricians, in predominantly urban prefectures, there is no evidence of a significant change in Gini trends in the *pre-period* (p-value  = 0.0943), and the trends do not differ between the *pre-* and the *post-period* (p-value  = 0.0584), whereas in other prefectures, there is a statistically significant decrease (i.e. improvement in Gini) in the *pre-period* (*β_1_* = −0.0042, p-value  = 0.0187) and the trend significantly differs between the *pre-* and the *post-period* (p-value  = 0.0343), becoming worse in the *post-period* (*β_3_* = 0.00536). Adjusted Gini Coefficients multiplied by n/n-1 (n is the number of municipalities in each prefecture) [37] were also used for a linear change-point regression procedure; however, the results did not change.

**Table 4:** Results of stratified analyses by the definition of OECD regional typology in linear change-point regression models for intra prefectural distributions using municipalities as the unit of analysis.

		Predominantly urban prefectures^a^			Others			
		(n = 13)			(n = 34)			
Effect		Estimate	SE^b^	p value	Estimate	SE^b^	p value	
**all physicians**								
β0	intercept	0.3192	0.02013	<.0001	0.2702	0.00968		<.0001
β1	year	−0.00510	0.00136	0.0003	−0.0019	0.00079		0.0201
β2	z^c^	−0.0206	0.00806	0.0124	−0.0177	0.00468		0.0002
β3	z^c^ •year	0.00645	0.00193	0.0012	0.00617	0.00112		<.0001
**pediatricians**								
β0	intercept	0.3542	0.01746	<.0001	0.3406	0.0127		<.0001
β1	year	−0.0042	0.00249	0.0943	−0.0042	0.00178		0.0187
β2	z^c^	−0.0245	0.01476	0.1008	−0.0249	0.01054		0.0192
β3	z^c^ •year	0.00677	0.00353	0.0584	0.00536	0.00252		0.0343

a: Miyagi, Saitama, Chiba, Tokyo, Kanagawa, Shizuoka, Aichi, Kyoto, Osaka, Hyogo, Nara, Hiroshima, and Fukuoka were defined as predominantly urban prefectures.

b: SE: standard error.

c:z: a function that equals 1 when year _ij_ > = 2004 and 0 otherwise.

**Figure 3:**
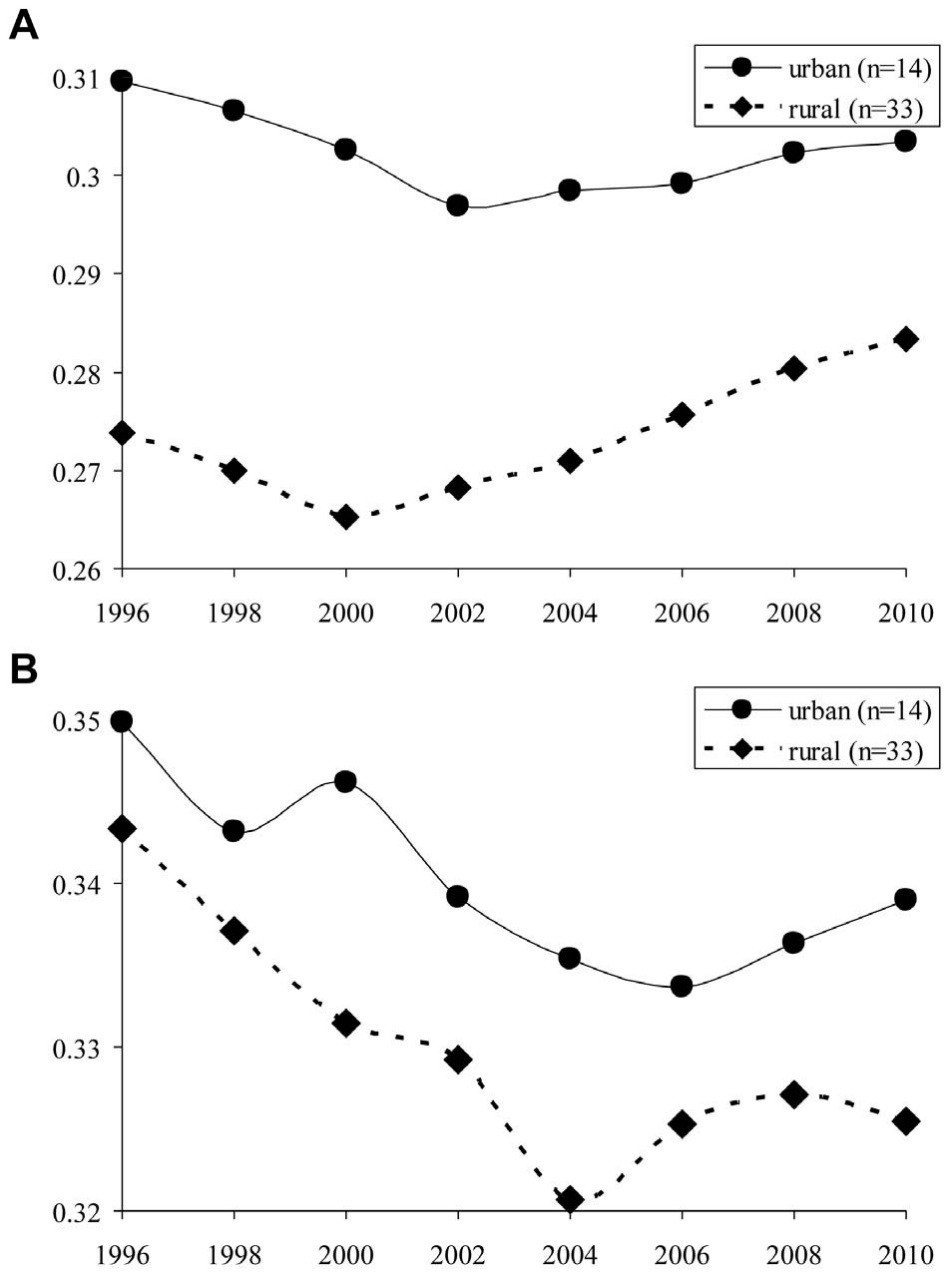
Figure 3-a; Mean of Gini coefficient of all physicians in intra-prefectural distributions; Prefectures were classified into urban and rural according to the metropolitan area codes. Figure 3-b; Mean of Gini coefficient of pediatricians in intra-prefectural distributions; Prefectures were classified into urban and rural according to the metropolitan area codes.

Table 5 shows the result from robustness check using the metropolitan area code as the urban/rural definition. A total of 14 prefectures (Hokkaido, Miyagi, Saitama, Chiba, Tokyo, Kanagawa, Niigata, Shizuoka, Aichi, Kyoto, Osaka, Hyogo, Hiroshima, and Fukuoka) include central cities for metropolitan areas and are defined as urban, and the remaining 33 are defined as rural. In 2010, 63.5% of the total population of Japan lived in urban areas, and the remaining of 36.5% lived in rural areas. The pattern of results was similar under this definition. The mean of the Gini coefficient in urban areas is higher for all physicians and pediatricians during study period (Figure 3-a and b). For all physicians, there were significant changes in trends for the worse between the *pre-* and the *post-period* both in urban and rural areas (p-values  = 0.0011 and <0.0001), whereas for pediatricians, there was a significant change in trends for the worse between the *pre-* and the *post-period* in rural areas (p-value  = 0.0156), but not in urban areas (p-value  = 0.1877). (Table 5).

**Table 5:** Results of stratified analyses by metropolitan areas in linear change-point regression models for intra-prefectural distributions using municipalities as the unit of analysis.

		Prefecture with central cities of major metropolitan areas ^a^				Other prefectures		
		(n = 14)				(n = 33)		
Effect		Estimate	SE^b^	p value	Estimate		SE^b^	p value
All physicians								
β0	intercept	0.3102	0.02038	<.0001	0.2726		0.009602	<.0001
β1	year	−0.00419	0.001263	0.0013	−0.00215		0.000845	0.0117
β2	z^c^	−0.01943	0.007474	0.0108	−0.01809		0.004998	0.0004
β3	z^c^ •year	0.006029	0.001787	0.0011	0.006344		0.001195	<.0001
Pediatricians								
β0	intercept	0.3489	0.0187	<.0001	0.3424		0.01257	<.0001
β1	year	−0.00291	0.002268	0.2029	−0.00477		0.001853	0.0106
β2	z^c^	−0.02027	0.01342	0.1342	−0.02665		0.01096	0.0158
β3	z^c^ •year	0.004256	0.003208	0.1877	0.006385		0.002621	0.0156

a: Hokkaido, Miyagi, Saitama, Chiba, Tokyo, Kanagawa, Niigata, Shizuoka, Aichi, Kyoto, Osaka, Hyogo, Hiroshima, and Fukuoka include central cities for metropolitan areas and are defined as urban.

b: SE: standard error.

c: Z: a function that equals 1 when year ij > = 2004 and 0 otherwise.

**Figure 4:**
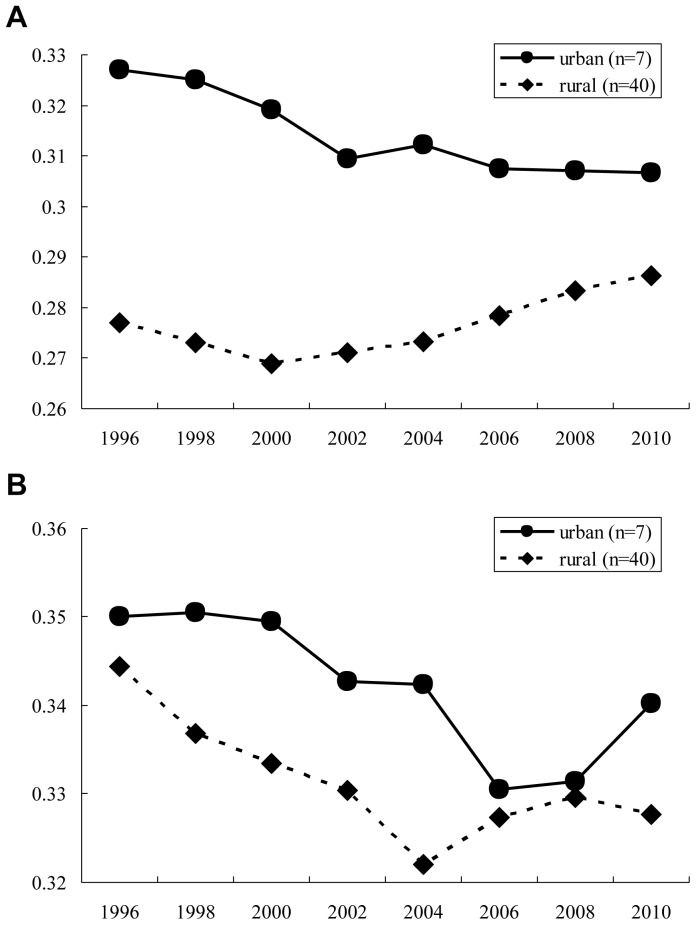
Figure 4-a Mean Gini coefficients of all physicians in intra-prefectural distributions: Prefectures were classified into urban and rural according to the population density. Figure 4-b Mean Gini coefficients of pediatricians in intra-prefectural distributions; Prefectures were classified into urban and rural according to the population density.

When population density was used as the urban/rural definition, a total of 7 prefectures (Saitama, Chiba, Tokyo, Kanagawa, Aichi, Osaka, and Fukuoka) are defined as urban and the remaining 40 are defined as rural. In 2010, 44.5% of the total population of Japan lived in urban areas, and the remaining 55.5% lived in rural areas. Namely, almost half of Japanese population lived in the above-mentioned 7 prefectures. The pattern of results was similar under this definition as well. The mean of the Gini coefficient in urban areas is higher for all physicians and pediatricians during study period (Figure 4-a and b). For all physicians, there were significant changes in trends for the worse between the *pre-* and the *post-period* both in urban and rural areas (p-values  = 0.0323 and <0.0001), whereas for pediatricians, there was a significant change in trends for the worse between the *pre-* and the *post-period* in rural areas (p-value  = 0.0064), but not in urban areas (p-value  = 0.6295). (Table 6).

**Table 6:** Results of stratified analyses by population density in linear change-point regression models for intra prefectural distributions using municipalities as the unit of analysis.

		Prefectures with population density			Prefectures with population density		
		> = 1000/km^2^ ^a^ (n = 7)			<1000/km2 (n = 40)		
Effect		Estimate	SE^b^	p value	Estimate	SE^b^	p value
**all physicians**							
β0	intercept	0.329	0.026	<.0001	0.2759	0.009609	<.0001
β1	year	−0.00587	0.001322	<.0001	−0.00221	0.000754	0.0036
β2	z^c^	−0.01104	0.007818	0.1646	−0.01979	0.004462	<.0001
β3	z^c^ •year	0.004126	0.001869	0.0323	0.006622	0.001067	<.0001
**pediatricians**							
β0	intercept	0.3516	0.01839	<.0001	0.3431	0.01179	<.0001
β1	year	−0.0023	0.002569	0.3752	−0.00455	0.00166	0.0065
β2	z^c^	−0.01255	0.0152	0.4131	−0.02689	0.009821	0.0066
β3	z^c^ •year	0.001765	0.003634	0.6295	0.006449	0.002348	0.0064

a: Saitama, Chiba, Tokyo, Kanagawa, Aichi, Osaka, and Fukuoka have population with more than 1000/km^2^.

b: SE: standard error.

c: Z: a function that equals 1 when year ij > = 2004 and 0 otherwise.

## Discussion

In this study, distributions of all physicians and pediatricians were examined over the period straddling the introduction of the new postgraduate medical training scheme in 2004. We note the following two major findings. First, the changes in trends in the geographic intra-prefectural distribution both all physicians and pediatricians differed significantly between the *pre-* and the *post-period*, with numbers trending upward after the introduction of the new postgraduate training scheme, which implies that the intra-prefectural distributions of physicians worsened. Second, in the stratified analyses by predominantly urban and other prefectures for pediatrics, there was a significant deterioration in trends in the *post-period* in other prefectures, though not in predominantly urban prefectures.

Previous quantitative analyses of physician distribution in Japan [4], [19]–[21] have not corroborated the public perception that physician distribution deteriorated after the launch of the new postgraduate training program. [38]–[40] For example, Ono K et al. [19] assessed the distribution of physicians and ophthalmologists before and after the new postgraduate training program using Gini coefficients for two time points: 1996 and 2006. They concluded that the geographical distribution of ophthalmologists and physicians did not worsen after the introduction of the new postgraduate training program. Similarly, Tanihara S et al. [20] examined geographic disparities in physician distribution using Gini coefficients for six time points between 1998 and 2008 and concluded that the Gini coefficient changed little during the study period. Toyabe S. [4] examined physician distribution using three measures: Gini coefficients, the Atkinson index and the Theil index, for six time points between 1996 and 2006. He concluded that the introduction of the new postgraduate training system had a profound effect on the maldistribution of physicians because the three measures of distribution remained at approximately the same level until 2002, deteriorated in 2004 and remained high in 2006. However, the differences between 2002 and 2004 were small (details were not provided in the paper), and only one time point after the launch of the new training system was examined in the study. Ehara (21) focused on pediatricians at two time points, 2002 and 2004, and compared the distribution of pediatricians using “Secondary Tier of Medical Care” (STM) as the spatial unit of analysis, which accounted for geographic location and travel route conditions and are supposed to be an independent administrative unit from a health service perspective. As measures of the distribution of pediatricians, minimum, percentile of 10, 20, 30, 50, 70, and 90, and maximum in the number of pediatricians were used. He concluded that the geographical distribution of pediatricians did not worsen after the launch of the new postgraduate training program. Our study also showed that the distribution of physicians did not worsen after the introduction of the new postgraduate training program, however, our study revealed that the intra-prefectural distribution of physicians worsened. It is possible that the deterioration of the intra-prefectural distribution of physicians resulted the poorer access to the medical care in some areas and led the public perception that physician distribution deteriorated after the launch of the new postgraduate training program.

Our study improves on the previous four studies because we used a longer period of observation after the launch of the new training system, which allowed us to have more power to detect the differences. We also showed statistically significant differences by employing proper statistical analysis. We believe that this study adds new evidence to the existing literature and heightens the debate about the impact of the postgraduate training program on physician distribution. The results of our study show that the intra-prefectural distribution of physicians worsened the *post-period*, which may reflect the impact of the new postgraduate program.

It is noteworthy that there was a significant change in trends of pediatrician intra-prefectural distribution between the *pre-* and the *post-period* in other prefectures but not in predominantly urban prefectures, which is also in agreement with the public perception that physician distribution became worse, especially in rural areas, after the launch of the new postgraduate training program in 2004. [38]–[40] Populations in rural areas are often disadvantaged in terms of health outcomes [41]–[43]. Poorer health outcomes in rural populations are attributable to many factors and access to health services is an important determinant of health outcomes for both ill-health treatment and preventive care [42]. This study showed that mean of the Gini coefficients of all physicians and pediatricians were higher in predominantly urban prefectures than in others, which means that the distribution of physicians was worse in predominantly urban prefectures than in others. Access to health care, however, is more difficult for rural residents in geographically remote territories, where services are widely dispersed at a low density because of greater distances to health services and limited transport options [44]–[47]. Lagarde M. and Blaauw D. stated that the geographical maldistribution of health workers exacerbates existing inequalities of access to basic health care and contributes to lower health outcomes for rural populations [48]. It is possible that the difference in trends between predominantly urban prefectures and others exacerbates rural-urban differentials in health outcomes for children, who are one of the most vulnerable subgroups. Further observation is needed to explore how this difference in trends affects the health status of the child population.

Our study has the following limitations. First, the Survey of Physicians, Dentists, and Pharmacologists does not include data on whether a physician works full time or part time. As a result, this analysis was based on an overall headcount, which might overestimate the number of physicians. In particular, the percentage of female physicians in pediatrics is high [49], as is the percentage of female physicians who work part time [50]. Therefore, it is likely that the numbers of practicing pediatricians were overestimated. Furthermore Nomura et al. found that female physicians were more likely than male physicians to work in university-affiliated hospitals. [51] Because these hospitals are more likely to dispatch physicians to rural areas, the gender balance of physicians in urban versus rural areas may exacerbate the shortage of clinicians practicing in rural areas. Because our data did not include information on physician gender, we were unable to analyze trends by gender. Second, information about work sites, such as clinics or hospitals, was not publicly available at the municipality level. Therefore, this variable could not be considered in the analysis, although the declining number of physicians working in hospitals has been seen as a problem in Japan [4], [19]–[21]. Third, we are only able to comment on whether the trends improved or worsened before and after the launch of the new postgraduate training program; we are unable to explain how the change in trends affects the health status of the population. No absolute optimal value for the Gini coefficient has been determined. Clearly, a longer observation period is needed to assess the ongoing impact of the new postgraduate training program on the health status of the population. Fourth, Gini coefficients were calculated for each prefecture. Therefore, this study considered the trend in distribution within each prefecture despite the ability of physicians and residents to migrate across prefectures. Fifth, urban-rural status is defined by prefecture level, but there are variations within each study unit (e.g., rural southern Illinois vs Chicago), which complicates the interpretation of prefecture-based findings. Sixth, we should note that the choice of spatial unit could lead to different conclusions regarding the pattern of geographic inequalities in the number of physicians. Lastly, the concentration of pediatricians did not necessarily worsen access to health care services. More specifically, at the beginning of the 21st century, the Japan Pediatric Society proceeded to concentrate the workforce of pediatricians into regional pediatric centers. The move was made in order to use them efficiently and to prevent the burnout of the physicians [52]. Ehara analyzed the time for patients to reach the regional pediatric centers and concluded that 90% of the child population in Japan would be able to arrive at the regional pediatric centers within one hour by car and 98% of child population would be able to do so within two hours; therefore, there would be only a limited child population who would be unable to access health care as a result of the concentration of the workforce of pediatricians [53].

Despite these limitations, we believe that our study contributes to the debate about the impact of the new postgraduate training program on inequality in the physician supply for the following reasons. First, we used time series data over a comparatively long period. Second, this is the first study to show statistically significant differences in the trends of physician distribution before and after the launch of the new training program. Third, our analyses reveal that a detailed and disaggregated approach by specialties is needed although previous studies considered only the overall number of physicians [1], [2], [4], [13], [23]. The stratified analyses by urban/rural status of physicians and pediatricians showed different trends. This result suggests that trends in the distribution of physicians vary according to specialty. Forth, we used three different definitions of urban/rural status because no standard definition of the term “rural” exists [30]–[33], and all analyses showed the similar results. Last, we also conducted the same analysis using “Secondary Titers of Medical Care” (STM) as the unit of analysis; however, the change of spatial unit did not greatly affect our conclusions. (Detailed results are shown in tables S1–S4).

## Conclusion

The changes in trends in the intra-prefectural distribution of physicians and pediatricians differed significantly before and after the launch of the new postgraduate training program, and our findings suggest an adverse impact of the new postgraduate training system. In pediatrics, changes in the Gini trend differed significantly before and after the launch of the new postgraduate training program in other prefectures, but not in predominantly urban prefectures. Further observation is needed to explore how this difference in trends affects the health status of the child population.

## Supporting Information

Table S1 **Results of linear change-point regression models for intra-prefectural distributions using Secondary Tier of Medical Care as the unit of analysis.**
(DOCX)

Table S2 **Results of stratified analyses by the definition of OECD regional typology in linear** **change-point regression models for intra prefectural distributions using Secondary Tier of Medical Care as the unit of analysis.**
(DOCX)

Table S3 **Results of stratified analyses by metropolitan areas in linear change-point regression models for intra-prefectural distributions using Secondary Tier of Medical Care as the unit of analysis.**
(DOCX)

Table S4 **Results of stratified analyses by population density in linear change-point regression models for intra-prefectural distributions using Secondary Tier of Medical Care as the unit of analysis.**
(DOCX)

## References

[1] Matsumoto M, Inoue K, Bowman R, Noguchi S, Toyokawa S, et al (2010) Geographical distributions of physicians in Japan and US: Impact of healthcare system on physician dispersal pattern. Health Policy 96: 255–261. 20236722 10.1016/j.healthpol.2010.02.012

[2] Matsumoto M, Inoue K, Farmer J, Inada H, Kajii E (2010) Geographic distribution of primary care physicians in Japan and Britain. Health Place 16: 164–166. 19733111 10.1016/j.healthplace.2009.07.005

[3] Stordeur S, Leonard C (2010) Challenges in physician supply planning: The case of Belgium. Hum.Resour.Health. 8: 28. 10.1186/1478-4491-8-28 PMC3017009 21138596

[4] Toyabe S (2009) Trend in geographic distribution of physicians in Japan. Int.J.Equity Health. 8: 5. 10.1186/1475-9276-8-5 PMC2662844 19257879

[5] Gorman D, Poole P, Scott SJ (2007) On the maldistribution of the medical workforce. Intern.Med.J. 37: 669–671. 10.1111/j.1445-5994.2007.01461.x 17894762

[6] Rosenthal MB, Zaslavsky A, Newhouse JP (2005) The geographic distribution of physicians revisited. Health Serv.Res. 40: 1931–1952. 10.1111/j.1475-6773.2005.00440.x PMC1361233 16336557

[7] Hann M, Gravelle H (2004) The maldistribution of general practitioners in England and Wales: 1974–2003. Br.J.Gen.Pract. 54: 894–898. PMC1326105 15588532

[8] Gravelle H, Sutton M (2001) Inequality in the geographical distribution of general practitioners in England and wales 1974–1995. J.Health Serv.Res.Policy 6: 6–13. 11219363 10.1258/1355819011927143

[9] Niel X (2001) Factors affecting regional distribution of french physicians. Cah.Sociol.Demogr.Med. 41: 141–172. 11490665

[10] Australian Medical Workforce Advisory Committee (AMWAC) (2000) Medical workforce planning in Australia. Aust.Health Rev. 23: 8–26. 11256274

[11] Alexander C (1998) Why doctors would stay in rural practice in the New England health area of new south Wales. Aust.J.Rural Health 6: 136–139. 9883107 10.1111/j.1440-1584.1998.tb00299.x

[12] McEldowney RP, Berry A (1995) Physician supply and distribution in the USA. J.Manag.Med. 9: 68–74. 10.1108/02689239510096839 10153506

[13] Kobayashi Y, Takaki H (1992) Geographic distribution of physicians in Japan. Lancet 340: 1391–1393. 1360099 10.1016/0140-6736(92)92569-2

[14] Yang BM, Huh J (1989) Physician distribution and health manpower policy in Korea. Asia.Pac.J.Public.Health. 3: 68–77, 85. 10.1177/101053958900300110 2719875

[15] Schwartz WB, Newhouse JP, Bennett BW, Williams AP (1980) The changing geographic distribution of board-certified physicians. N.Engl.J.Med. 303: 1032–1038. 10.1056/NEJM198010303031803 7421890

[16] Japan Medical Association Maldistribution of physician (in Japanese) Available at http://www.med.or.jp/nichinews/n241220l.html. Accessed on January 19 2013.

[17] The Kochi Shimbun.Shortage of physicians, still exsists even after the advent of a new administration. Available at http://www.kochinews.co.jp/?&nwSrl=296591&nwIW=1&nwVt=knd [in Japanese]. Accessed on January 19 2013.

[18] Ministry of Health, Labour and Welfare Outline of postgraduate clinical training. Conference paper presented at working group on postgraduate clinical training. Available at http://www.mhlw.go.jp/shingi/0106/s0601-1.html#s2-1 [in Japanese]. Accessed on April 13 2012.

[19] Ono K, Hiratsuka Y, Murakami A (2010) Geographical distribution of ophthalmologists before and after the new postgraduate training program in Japan. Ophthalmic Epidemiol. 17: 125–130. 10.3109/09286581003624988 20302434

[20] Tanihara S, Kobayashi Y, Une H, Kawachi I (2011) Urbanization and physician maldistribution: A longitudinal study in Japan. BMC Health Serv.Res. 11: 260. 10.1186/1472-6963-11-260 PMC3204230 21982582

[21] Ehara A (2007) Situation in pediatricians before and after the launch of the new postgraduate training program in 2004–inequality in number of pediatricians did not worse after he launch of the program compared to before the launch [in Japanese]. Nihon Ishikai Zasshi 136: 1804–1808.

[22] Association to support the certification of dease due to overwork of Dr.Nakahara. Incident of death due to overwork of dr.nakahara. Available at http://www5f.biglobe.ne.jp/~nakahara/. Accessed on 07/12 2002.

[23] Matsumoto M, Inoue K, Noguchi S, Toyokawa S, Kajii E (2009) Community characteristics that attract physicians in Japan: A cross-sectional analysis of community demographic and economic factors. Hum.Resour.Health. 7: 12. 10.1186/1478-4491-7-12 PMC2649032 19226450

[24] Matsumoto M, Inoue K, Bowman R, Kajii E (2010) Self-employment, specialty choice, and geographical distribution of physicians in Japan: A comparison with the United States. Health Policy 96: 239–244. 20223549 10.1016/j.healthpol.2010.02.008

[25] Ministry of Health, Labour and Welfare. Survey of physicians, dentists, and pharmacologist. Available at http://www.e-stat.go.jp/SG1/estat/NewList.do?tid=000001030962 [in Japanese]. Accessed on April 13 2012.

[26] Ministry of Health, Labour and Welfare. Survey summary. Available at http://www.mhlw.go.jp/toukei/saikin/hw/ishi/10/dl/tyousa.pdf [in Japanese]. Accessed on April 13 2012.

[27] Shimada N, Kondo T (2004) Estimated registry rate using individual data of survey of physicians, dentists, and pharmacologists [in Japanese]. Japanese Journal of Public Health 51: 117–132. 15058102

[28] Ministry of Internal Affairs and Communications.Basic resident registers. Available at http://www.e-stat.go.jp/SG1/estat/GL08020102.do?_toGL08020102_&tclassID=000001028704&cycleCode=7&requestSender=estat [in Japanese]. Accessed on February 2 2013.

[29] Organization for Economic Cooperation and Development (OECD). OECD regional typology. Available at http://www.oecd.org/gov/regional-policy/OECD_regional_typology_Nov2012.pdf. Accessed on July 20th 2013.

[30] Matsumoto M, Inoue K, Kajii E (2010) Definition of “rural” determines the placement outcomes of a rural medical education program: Analysis of jichi medical university graduates. J.Rural Health 26: 234–239. 20633091 10.1111/j.1748-0361.2010.00286.x

[31] Rabinowitz HK, Paynter NP (2000) The role of the medical school in rural graduate medical education: Pipeline or control valve? J.Rural Health 16: 249–253. 11131769 10.1111/j.1748-0361.2000.tb00468.x

[32] Rabinowitz HK, Diamond JJ, Markham FW, Wortman JR (2008) Medical school programs to increase the rural physician supply: A systematic review and projected impact of widespread replication. Acad.Med. 83: 235–243. 10.1097/ACM.0b013e318163789b 18316867

[33] Rabinowitz HK, Petterson S, Boulger JG, Hunsaker ML, Diamond JJ, et al (2012) Medical school rural programs: A comparison with international medical graduates in addressing state-level rural family physician and primary care supply. Acad.Med. 87: 488–492. 10.1097/ACM.0b013e3182488b19 22361802

[34] Odisho AY, Fradet V, Cooperberg MR, Ahmad AE, Carroll PR (2009) Geographic distribution of urologists throughout the United States using a county level approach. J.Urol. 181: 760–5; discussion 765–6. 10.1016/j.juro.2008.10.034 19091334

[35] Sakai R (2011) Relationship between prevalence of childhood obesity in 17-year-olds and socioeconomic and environmental factors: Prefecture-level analysis in Japan. Asia.Pac.J.Public.Health. 10.1177/1010539511416347 21807624

[36] Bureau of Statistics Ministry of Internal Affairs and Communications. Major metropolitan areas, metropolitan areas, and their central cities and surrounding municipalities, description of the area classification used in the statistical tables, census 2005. Available at http://www.stat.go.jp/data/kokusei/2005/users/kubun.htm#pos4 [in Japanese]. Accessed on July 10 2012.

[37] Deltas G (2003) The small-sample bias of the gini coefficient: Results and implications for empirical research. Review of Economics and Statistics 85: 226–234.

[38] Science Counsel of Japan. Those underlying the maldistribution of physicians. Available at http://www.scj.go.jp/ja/info/kohyo/pdf/kohyo-20-t39-2.pdf. [in Japanese]. Accessed on Aug 6th 2013.

[39] Ogawa A. New postgraduate training program -light and shadow-. Available at http://dl.med.or.jp/dl-med/nichikara/isei/isei2010_6.pdf [in Japanese]. Accessed on July 13 2013.

[40] Ministry of Health, Labor and Welfare. Review of the postgraduate training program [in Japanese]. Available at http://www.mhlw.go.jp/seisaku/2009/08/04.html. Accessed on February 17 2013.

[41] Matsumoto M, Bowman R, Worley P (2012) A guide to reporting studies in rural and remote health. Rural Remote Health. 12: 2312. 22950574

[42] Smith KB, Humphreys JS, Wilson MG (2008) Addressing the health disadvantage of rural populations: How does epidemiological evidence inform rural health policies and research? Aust.J.Rural Health 16: 56–66. 18318846 10.1111/j.1440-1584.2008.00953.x

[43] Ricketts TC (2000) The changing nature of rural health care. Annu.Rev.Public Health 21: 639–657. 10884968 10.1146/annurev.publhealth.21.1.639

[44] Launoy G, Le Coutour X, Gignoux M, Pottier D, Dugleux G (1992) Influence of rural environment on diagnosis, treatment, and prognosis of colorectal cancer. J.Epidemiol.Community Health 46: 365–367. 1431708 10.1136/jech.46.4.365 PMC1059601

[45] Dejardin O, Bouvier AM, Herbert C, Velten M, Buemi A, et al (2005) Social and geographic disparities in access to reference care site for patients with colorectal cancer in France. Br.J.Cancer 92: 1842–1845. 15886707 10.1038/sj.bjc.6602571 PMC2361758

[46] Taylor HA, Hughes GD, Garrison RJ (2002) Cardiovascular disease among women residing in rural America: Epidemiology, explanations, and challenges. Am.J.Public Health 92: 548–551. 11919049 10.2105/ajph.92.4.548 PMC1447114

[47] Wainer J, Chesters J (2000) Rural mental health: Neither romanticism nor despair. Aust.J.Rural Health 8: 141–147. 11249401 10.1046/j.1440-1584.2000.00304.x

[48] Lagarde M, Blaauw D (2009) A review of the application and contribution of discrete choice experiments to inform human resources policy interventions. Hum.Resour.Health. 7: 62. 10.1186/1478-4491-7-62 PMC2724490 19630965

[49] Ministry of Health, Labour and Welfare. Survey summary; 1. Physician. Available at http://www.mhlw.go.jp/toukei/saikin/hw/ishi/10/dl/kekka_1.pdf [in Japanese]. Accessed on April 13 2012.

[50] McMurray JE, Heiligers PJ, Shugerman RP, Douglas JA, Gangnon RE, et al (2005) Part-time medical practice: Where is it headed? Am.J.Med. 118: 87–92. 10.1016/j.amjmed.2004.11.005 15639215

[51] Nomura K, Yano E, Mizushima S, Endo H, Aoki M, et al (2008) The shift of residents from university to non-university hospitals in Japan: A survey study. J.Gen.Intern.Med. 23: 1105–1109. 10.1007/s11606-008-0644-7 PMC2517916 18612753

[52] Japan Pediatric Society, & Investigative Committee for Pediatric Care Structure. The reform of pediatric care structure. Available at http://jpsmodel.umin.jp/introduction.html [in Japanese]. Accessed on June 1st 2013.

[53] Ehara A (2008) Hokkaido prefecture government showed a plan of concentration of pediatric emergency centers [in Japanese]. 112: 879–882.

