# Supplementary material for: The Impact of Japan's 2004 Postgraduate Training Program on Intra-Prefectural Distribution of Pediatricians in Japan
Source: PLoS One. 2013 Oct 30;8(10):e77045. doi: 10.1371/journal.pone.0077045 (PMC3813669; doi:10.1371/journal.pone.0077045)
Supplement: Table S1 — Results of linear change-point regression models for intra-prefectural distributions using Secondary Tier of Medical Care as the unit of analysis. (DOCX) [file pone.0077045.s001.docx]

| Effect |  | Estimate | SE^a^ | p value |
| --- | --- | --- | --- | --- |
| all physician |  |  |  |  |
| β0 | intercept | 0.1573 | 0.006969 | <.0001 |
| β1 | year | -0.00202 | 0.000585 | 0.0006 |
| β2 | z^b^ | -0.01868 | 0.004178 | <.0001 |
| β3 | z^b^ •year | 0.004317 | 0.000827 | <.0001 |
|  |  |  |  |  |
| pediatrician |  |  |  |  |
| β0 | intercept | 0.1871 | 0.008294 | <.0001 |
| β1 | year | -0.00475 | 0.001296 | 0.0003 |
| β2 | z^b^ | -0.02455 | 0.007665 | 0.0015 |
| β3 | z^b^ •year | 0.005532 | 0.001832 | 0.0027 |

| a: SE: standard error |
| --- |
| b: Z: a function that equals 1 when year _ij_ > 2004 and 0 otherwise |
| Table S1: Results of linear change-point regression models for intra-prefectural distributions using Secondary Tier of Medical Care as the unit of analysis |
