# Supplementary material for: The Impact of Japan's 2004 Postgraduate Training Program on Intra-Prefectural Distribution of Pediatricians in Japan
Source: PLoS One. 2013 Oct 30;8(10):e77045. doi: 10.1371/journal.pone.0077045 (PMC3813669; doi:10.1371/journal.pone.0077045)
Supplement: Table S2 — Results of stratified analyses by the definition of OECD regional typology in linear change-point regression models for intra prefectural distributions using Secondary Tier of Medical Care as the unit of analysis. (DOCX) [file pone.0077045.s002.docx]

|  |  | Predominantly urban prefectures | | |  | Others | | |
| --- | --- | --- | --- | --- | --- | --- | --- | --- |
|  |  | (n=13) | | |  | (n=34) | | |
| **Effect** | | Estimate | SE^a^ | p value |  | Estimate | SE^a^ | p value |
| **all physicians** | | | | | | | | |
| β0 | intercept | 0.1674 | 0.02001 | <.0001 |  | 0.1506 | 0.00588 | <.0001 |
| β1 | year | -0.0036 | 0.00096 | 0.0003 |  | -0.0014 | 0.00065 | 0.0308 |
| β2 | z^b^ | -0.0081 | 0.00569 | 0.1566 |  | -0.0168 | 0.00386 | <.0001 |
| β3 | z^b^ •year | 0.00271 | 0.00136 | 0.0490 |  | 0.00493 | 0.00092 | <.0001 |
| **pediatricians** | | | | | | | | |
| β0 | intercept | 0.1871 | 0.01818 | <.0001 |  | 0.187 | 0.0093 | <.0001 |
| β1 | year | -0.0051 | 0.00204 | 0.0152 |  | -0.0046 | 0.00162 | 0.0046 |
| β2 | z^b^ | -0.0261 | 0.01207 | 0.0335 |  | -0.024 | 0.00957 | 0.0129 |
| β3 | z^b^ •year | 0.00647 | 0.00289 | 0.0276 |  | 0.00518 | 0.00229 | 0.0246 |

a: SE : standard error

b:z: a function that equals 1 when year _ij_ >= 2004 and 0 otherwise

Table S2. Results of stratified analyses by the definition of OECD regional typology in linear change-point regression models for intra prefectural distributions using Secondary Tier of Medical Care as the unit of analysis
