# Supplementary material for: The Impact of Japan's 2004 Postgraduate Training Program on Intra-Prefectural Distribution of Pediatricians in Japan
Source: PLoS One. 2013 Oct 30;8(10):e77045. doi: 10.1371/journal.pone.0077045 (PMC3813669; doi:10.1371/journal.pone.0077045)
Supplement: Table S3 — Results of stratified analyses by metropolitan areas in linear change-point regression models for intra-prefectural distributions using Secondary Tier of Medical Care as the unit of analysis. (DOCX) [file pone.0077045.s003.docx]

|  |  | Prefectures with central cities of major metropolitan areas (n=14) | | |  | Other prefectures (n=33) | | |
| --- | --- | --- | --- | --- | --- | --- | --- | --- |
|  |  |  |  |  |  |  |  |  |
| Effect | | Estimate | SE^a^ | p value |  | Estimate | SE^a^ | p value |
| all physicians | |  |  |  |  |  |  |  |
| β0 | intercept | 0.1678 | 0.01818 | <.0001 |  | 0.1499 | 0.006208 | <.0001 |
| β1 | year | -0.00326 | 0.000849 | 0.0002 |  | -0.00149 | 0.000705 | 0.0358 |
| β2 | z^b^ | -0.01236 | 0.00502 | 0.0156 |  | -0.01522 | 0.004169 | 0.0003 |
| β3 | z^b^ •year | 0.00351 | 0.0012 | 0.0043 |  | 0.00466 | 0.000996 | <.0001 |
|  |  |  |  |  |  |  |  |  |
| pediatricians | |  |  |  |  |  |  |  |
| β0 | intercept | 0.1904 | 0.01592 | <.0001 |  | 0.1856 | 0.009844 | <.0001 |
| β1 | year | -0.00445 | 0.001754 | 0.0127 |  | -0.00488 | 0.001695 | 0.0044 |
| β2 | z^b^ | -0.02294 | 0.01038 | 0.0295 |  | -0.02523 | 0.01003 | 0.0125 |
| β3 | z^b^ •year | 0.00482 | 0.002481 | 0.055 |  | 0.005834 | 0.002397 | 0.0157 |

| a: SE: standard error |
| --- |
| b: Z: a function that equals 1 when year ij >= 2004 and 0 otherwise  Table S3: Results of stratified analyses by metropolitan areas in linear change-point regression models for intra-prefectural distributions using Secondary Tier of Medical Care as the unit of analysis |
