# Supplementary material for: The Impact of Japan's 2004 Postgraduate Training Program on Intra-Prefectural Distribution of Pediatricians in Japan
Source: PLoS One. 2013 Oct 30;8(10):e77045. doi: 10.1371/journal.pone.0077045 (PMC3813669; doi:10.1371/journal.pone.0077045)
Supplement: Table S4 — Results of stratified analyses by population density in linear change-point regression models for intra-prefectural distributions using Secondary Tier of Medical Care as the unit of analysis. (DOCX) [file pone.0077045.s004.docx]

|  |  | prefecture with population density | | |  | prefecture with population density | | |
| --- | --- | --- | --- | --- | --- | --- | --- | --- |
|  |  | >=1000/km^2^ (n=7) | | |  | <1000/km2 (n=40) | | |
| **Effect** | | Estimate | SE^a^ | p value |  | Estimate | SE^a^ | p value |
| **all physicians** | | | | | | | | |
| β0 | intercept | 0.183 | 0.03169 | 0.0012 |  | 0.1504 | 0.006025 | <.0001 |
| β1 | year | -0.00395 | 0.001277 | 0.0033 |  | -0.00168 | 0.000608 | 0.0063 |
| β2 | z^b^ | -0.00605 | 0.007554 | 0.4273 |  | -0.01582 | 0.003599 | <.0001 |
| β3 | z^b^ •year | 0.002098 | 0.001806 | 0.2512 |  | 0.004705 | 0.00086 | <.0001 |
| **pediatricians** | | | | | | | | |
| β0 | intercept | 0.1922 | 0.02389 | 0.0002 |  | 0.1861 | 0.008932 | <.0001 |
| β1 | year | -0.00274 | 0.002396 | 0.2593 |  | -0.0051 | 0.00146 | 0.0006 |
| β2 | z^b^ | -0.02282 | 0.01418 | 0.1143 |  | -0.02485 | 0.00864 | 0.0043 |
| β3 | z^b^ •year | 0.002351 | 0.003389 | 0.4914 |  | 0.006089 | 0.002065 | 0.0035 |
| a: SE: standard error  b: Z: a function that equals 1 when year ij >= 2004 and 0 otherwise  Table S4. Results of stratified analyses by population density in linear change-point regression models for intra-prefectural distributions using Secondary Tier of Medical Care as the unit of analysis | | | | | | | | |
